# TRAF2 and NCK interacting kinase: a novel regulator of integrin α_IIb_β_3_ signaling in platelets

**DOI:** 10.1016/j.rpth.2025.103204

**Published:** 2025-10-06

**Authors:** Lily J. Bull, Scott Spencer, Ronit Bedi, Rebecca Lewis, Lisa-Marie Holbrook

**Affiliations:** 1 Department of Comparative Biomedical Sciences, School of Veterinary Medicine, University of Surrey, Guildford, UK; 2 School of Biosciences, University of Surrey, Guildford, UK

**Keywords:** blood platelets, cytoskeleton, integrins, protein serine-threonine kinases, TNIK

## Abstract

**Background:**

TRAF2 and NCK interacting kinase (TNIK) is a serine/threonine kinase member of the germinal center kinase family. Previously, in other cell types, TNIK has been shown to interact with key cytoskeletal regulatory proteins, to regulate F-actin distribution, cell polarization, and neuronal cell arborization. Proteomic studies have demonstrated TNIK is present in platelets; however, no further studies have explored if TNIK plays a role in the regulation of platelet function.

**Objectives:**

We sought to investigate the distribution of TNIK in platelets and characterize a potential role for TNIK in platelet function.

**Methods:**

The importance of platelet TNIK was explored using 2 structurally distinct TNIK inhibitors (KY-05009 and NCB-0846) in aggregometry, assays of granule secretion, calcium mobilization and thrombus formation, and using confocal microscopy and co-immunoprecipitation.

**Results:**

TNIK inhibition diminished platelet responses, and thrombus formation under arterial shear. Furthermore, TNIK regulated platelet adhesion and spreading on fibrinogen and impaired clot retraction due to reduced integrin α_IIb_β_3_ activation. Co-immunoprecipitation studies demonstrated that TNIK bridges interactions between the actin cytoskeleton and integrin α_IIb_β_3_ through direct associations with focal adhesion kinase, paxillin, kindlin-3, filamin A, Rap2a, gelsolin and actin.

**Conclusion:**

In summary, we confirm TNIK is present in platelets and is important for platelet function, and thrombus formation. For the first time, we show that platelet TNIK is able to associate with key cytoskeletal regulators, linking its signaling to integrin α_IIb_β_3_ function. Future studies will focus on exploring a potential role of TNIK in the regulation of thrombosis and hemostasis *in vivo* to assess TNIK’s potential as a novel antithrombotic target.

## Essentials

• TRAF2 and NCK interacting kinase (TNIK) is a serine/threonine kinase with no currently known role in platelets.
• TNIK function was explored in platelets using 2 structurally distinct inhibitors.
• Inhibition of TNIK reduces markers of platelet integrin signaling.
• TNIK may be a novel antiplatelet target.

## 1 Introduction

Platelets are critical regulators of hemostasis, which, following damage to the vascular endothelium, accumulate at the site of injury through binding to subendothelial matrix proteins such as collagen. Exposure to matrix proteins activates platelets through binding to platelet cell surface receptors, which triggers complex intracellular signaling leading to granule release, activation of integrin αIIbβ3, and platelet aggregation. These events are underpinned by changes to the actin cytoskeleton; however, the exact regulatory mechanisms and proteins involved are still emerging.

Platelet activation and shape change is tightly controlled by a host of regulatory kinases. Proteomic analyses of human platelets have identified >100 serine/threonine kinases; however, the roles of many of these kinases in platelet function have not been explored [1]. In the present study, we aimed to characterize the role of 1 such kinase, TRAF2 and NCK interacting kinase (TNIK), in platelet function and signaling.

TNIK is a serine/threonine kinase of the germinal center kinase family, which has been shown to regulate cytoskeletal changes in various cell types through a range of effector proteins [2]. TNIK was first shown to regulate F-actin distribution in kidney epithelial cells, where overexpression of TNIK resulted in impaired cell spreading and attachment. In these cells, TNIK regulated F-actin through phosphorylation of an actin-binding protein gelsolin [2]. Similarly, in intestinal epithelial cells, TNIK has been shown to regulate intestinal brush border formation, through phosphorylation of the actin-cytoskeleton linker protein Ezrin [3]. TNIK has been shown to colocalize with the small GTP-binding protein Rap2a, and this interaction aids TNIK in its translocation to the cytoskeleton to support its role as a regulator of cytoskeletal changes [4]. In neuronal cells, TNIK signaling is regulated by the E3 ubiquitin ligase Nedd4-1, which complexes Rap2a resulting in Rap2a ubiquitination. This change in Rap2a inhibits TNIK signaling, leading to a promotion of dendrite growth through alleviation of the TNIK-mediated cytoskeletal repression [5].

TNIK has been widely investigated in preclinical models, in a variety of disease contexts, including cancer [[6], [7], [8], [9], [10], [11], [12], [13], [14], [15]], cardiovascular disease [16], neurodegenerative disease [[17], [18], [19]], and fibrotic disorders [20,21]. A range of platelet proteome studies have previously identified TNIK in platelets [[22], [23], [24], [25]]; however, no further investigation into TNIK’s role in platelets has been reported. In this study, we show, for the first time, the localization of TNIK in human platelets and through TNIK inhibition, and that TNIK plays a significant role in platelet aggregation, granule secretion, and calcium mobilization. We observed impaired platelet adhesion and spreading on immobilized fibrinogen, aberrant clot retraction, and impaired thrombus formation following TNIK inhibition. Moreover, TNIK clusters with proteins that are crucial for cytoskeletal modulation and integrin function. We hypothesized that, in platelets, TNIK signaling induces changes to these cytoskeletal regulatory proteins, which directly interact with integrin α_IIb_β_3_ leading to changes in integrin α_IIb_β_3_ activity to influence platelet aggregation, spreading, and thrombus growth.

## 2 Materials

Cultrex bovine collagen type I was purchased from BioTechne and equine tendon collagen was from Helena Biosciences. Fibrinogen from human plasma, apyrase, bovine thrombin, 3,3′-dihexyloxacarbocyanine iodide (DIOC6), indomethacin, integrilin, and protease-free bovine serum albumin were purchased from Sigma Aldrich. Thrombin receptor activator peptide (TRAP)-6 was obtained from Bachem, collagen-related peptide (CRP)-A was purchased from P-Plus Products. U46619, prostaglandin I_2_ (PGI_2_), NCB-0846, KY-05009, and protease inhibitor cocktail were purchased from Cambridge Biosciences. Alexa Fluor 568 conjugated Phalloidin and ProLong Gold antifade mounting media were purchased from Invitrogen. ATP-Glo Bioluminometric Assay kit was purchased from Biotium, protein A/G beads were from Santa Cruz Biotechnology, and Fura-8 AM was from AAT Bioquest.

Phospho-(Ser)-protein kinase C (PKC) substrate, TNIK, Phospho-focal adhesion kinase (FAK; Y397), paxillin, phospho-(Y118)-paxillin, kindlin-3, Rap2a, gelsolin antirabbit immunoglobulin G (IgG) Dylight 800-conjugate, anti-rabbit IgG horseradish peroxidase conjugate and anti-mouse IgG Dylight 680-conjugated antibodies were purchased from Cell Signalling Technology. Total FAK antibody was purchased from Biorbyt. Anti-α-tubulin and anti-β-actin antibodies were purchased from Sigma Aldrich. PAC-1 fluorescein isothiocyanate-conjugated, phycoerythrin (PE)-conjugated mouse anti-human CD62P antibodies and annexin V PE conjugate were purchased from BD Biosciences. Goat anti-rabbit IgG (H+L) highly cross-absorbed Alexa Fluor 488, and goat anti-mouse IgG (H+L) Alexa Fluor 647 secondary antibodies were purchased from Invitrogen. TNIK (30906-1-AP) antibody for co-immunoprecipitation was purchased from Proteintech.

### 2.1 Platelet preparation

Human washed platelets were prepared as previously described [26], and platelets were resuspended in Tyrodes-HEPES buffer (134 mM NaCl, 0.34 mM Na_2_HPO_4_, 2.9 mM KCl, 12 mM NaHCO_3_, 20 mM HEPES, 1 mM MgCl_2_, 5 mM glucose, pH 7.3) to desired cell density and rested for 30 minutes prior to use.

### 2.2 Platelet aggregation assays

Washed platelets (4 × 10^8^ cells/mL) were preincubated in the presence of vehicle (dimethylsulfoxide [DMSO], 0.5% v/v), KY-05009 (1-10 μM) or NCB-0846 (1-10 μM) for 5 minutes prior to stimulation with collagen (2.5 μg/mL), TRAP-6 (5 μM), or U46619 (1 μM). Platelet aggregation was monitored for 3 minutes using light transmission aggregometry under stirring conditions (1200 rpm).

### 2.3 Calcium mobilization measurement

Platelet-rich plasma (PRP) was loaded with FURA-8 AM (0.5 μM) before centrifugation at 300*g*, for 20 minutes in the presence of PGI_2_ (2.8 μM). Platelets were resuspended in Tyrodes-HEPES buffer to a cell density of 4 × 10^8^ cells/mL. Platelets were incubated in the presence of vehicle (DMSO, 0.5% v/v) or NCB-0846 (1-10 μM) for 5 minutes prior to stimulation with CRP (0.5 μg/mL). Fluorescence measurements with excitation at 340 and 360 nm and emission at 520 and 540 nm were recorded using a CLARIOstar plate reader (BMG Labtech).

### 2.4 Platelet granule secretion, annexin V binding, and integrin α_IIb_β_3_ activation measurements

Integrin α_IIb_β_3_ activation, P-selectin, and annexin V surface expression were measured using PAC-1 antibody (1:250 dilution), CD62P (P-selectin) antibody (1:500 dilution), or annexin V fluorescent conjugate (1:250 dilution), respectively. Washed platelets (2 × 10^8^ cells/mL) were diluted in HEPES-buffered saline (10 mM HEPES, 1 mM NaCl, 1 mM MgSO_4_, 1 mM CaCl_2_) and stimulated with CRP (0.5 μg/mL), TRAP-6 (5 μM) or U46619 (1 μM) for 20 minutes. Platelets were fixed by dilution in formylsaline (0.2% v/v), data for 10,000 gated events were collected and analyzed using a BD Accuri C6 plus flow cytometer and C6 plus software (version 1.0.264.2; BD Biosciences).

Adenosine triphosphate (ATP) secretion from dense granules was measured in washed platelets (4 × 10^8^ cells/mL) using an ATP-Glo Bioluminometric Assay (Biotium) as per manufacturers instruction. Platelets were preincubated in the presence of vehicle (DMSO, 0.5% v/v) or KY-05009 (1-10 μM) for 5 minutes prior to stimulation with CRP (1 μg/mL). Luminescence was measured using a CLARIOstar plate reader.

### 2.5 Platelet spreading

Washed human platelets (4 × 10^7^ cells/mL) were spread on collagen (100 μg/mL) or fibrinogen (100 μg/mL) coated coverslips in the presence of vehicle (DMSO, 0.5% v/v) or KY-05009 (10 μM). After 45 minutes, coverslips were washed and fixed with paraformaldehyde (0.2% v/v) and permeabilized with Triton X-100 (0.2%, v/v). F-actin was stained with Alexa Fluor 568 phalloidin (1:100 dilution), and coverslips were mounted onto glass slides using using ProLong Gold Antifade mounting media. Platelets were visualized at 40× magnification using a Nikon 40X/0.65 Ph2 DL WD 6.5 objective and an ECLIPSE Ts2-FL Diascopic and Epi-fluorescence microscope.

### 2.6 Human clot retraction

PRP was incubated for 5 minutes with vehicle (DMSO, 0.5% v/v), or KY-05009 (100 μM). Red blood cells were added for contrast (0.5% v/v), and clot retraction was induced with thrombin (1 unit/mL). Clot formation was monitored for 30 minutes and images captured. Clot size was determined using ImageJ.

### 2.7 Immunofluorescence

PRP was incubated with integrilin (4 μM) to block platelet aggregation then platelets were stimulated with CRP (1 μg/mL), TRAP-6 (10 μM), or U46619 (5 μM) for 5 minutes and fixed with paraformaldehyde (4% v/v). Platelets were pelleted at 950*g* for 10 minutes, then washed twice in ACD (in phosphate-buffered saline [PBS]), then resuspended in bovine serum albumin [BSA]-PBS (1% w/v), and adhered on poly-L-lysine–coated coverslips. Slides were blocked and permeabilized (TritonX-100 [0.2% v/v], filtered goat serum [2% v/v], and protease-free BSA [1% w/v]), and then anti-α-tubulin (1:200 dilution) and anti-TNIK antibodies (1:100 dilution) were added overnight at 4 °C. Coverslips were washed with PBS (3 times), before F-actin was stained with Alexa Fluor 568 phalloidin (1:100 dilution) and the addition of fluorescently conjugated secondary anti-mouse Alexa Fluor 647 (1:250 dilution) and anti-rabbit Alexa Fluor 488 (1:1000 dilution) antibodies for 90 minutes. Coverslips were washed, then fixed with paraformaldehyde-PBS (4% v/v), and then mounted using ProLong Gold Antifade mounting media. Images were captured using a Zeiss LSM980 confocal microscope using a Plan-Apochromat 63X/1.4 oil differential interference contrast objective. Pearson's correlation coefficient was used to assess protein colocalization as described previously [27].

### 2.8 Immunoprecipitation

Platelets (8 × 10^8^ cells/mL) were prepared under non-aggregating conditions by the addition of indomethacin (10 μM), EGTA (1 mM), and apyrase (2 units/mL) and then stimulated with Tyrodes-HEPES or collagen (25 μg/mL) for 3 minutes under stirred conditions. Cells were pelleted at 1500*g*, for 5 minutes and lysed in NP40 lysis buffer (150 mM NaCl, 10 mM Tris, 5 mM EDTA, 1% [v/v] NP40, pH 7.3). Platelet lysate was precleared with protein A/G beads for 60 minutes at 4 °C, then incubated with TNIK antibody (1 μg) for 4 hours and then immune-protein complexes captured with protein A/G beads. Beads were washed 3 times in 0.5% (v/v) NP40 lysis buffer and then once in Tris-buffered saline-Tween (TBS-T, 20 mM Tris, 150 mM NaCl, 0.1% [v/v] Tween-20, pH 7.6) prior to separation by sodium dodecyl sulfate–polyacrylamide gel electrophoresis (SDS-PAGE).

### 2.9 SDS-PAGE and immunoblotting

Proteins were separated by reducing SDS-PAGE using 4% to 20% Novex Tris-Glycine gels alongside prestained protein standards. Gels were transferred to polyvinylidene fluoride membrane and blocked with SuperBlock blocking buffer. Primary antibodies were added overnight at 4 °C and following washes with TBS-T, membranes were visualized with either fluorescently conjugated or horseradish peroxidase–conjugated secondary antibodies using an Odyssey Fc Imager (LI-COR). To re-probe membranes, membranes were stripped using a stripping buffer containing betamercaptoethanol (0.1 M) for 120 minutes.

### 2.10 Thrombus formation under arterial shear

Thrombus formation was measured using an ExiGo Microfluidic perfusion system (Cellix). Briefly, Vena8 Fluoro+ Biochips were coated with collagen (100 μg/mL) overnight at 4 °C and blocked with protease-free BSA (10 μg/mL) for 60 minutes at 37 °C. Whole blood was labeled for 60 minutes with 3,3′-dihexyloxacarbocyanine iodide (DIOC6; 1 μM), then incubated with vehicle (DMSO, 1% v/v) or KY-05009 (10, 50, or 100 μM) for 5 minutes prior to perfusion over channels at 20 dynes/cm^2^. Thrombus formation was monitored for 10 minutes using a Nikon 40X/0.65 Ph2 DL WD 6.5 objective and an ECLIPSE Ts2-FL Diascopic and Epi-fluorescence microscope. Fluorescence data were analyzed using NIKON NIS Elements AR (version 5.12.03).

### 2.11 Ethical approval and data analysis

Blood collection from consenting healthy volunteers was approved by the University of Surrey local ethical review panel (FHMS 22-23 221 EGA) in accordance with the Declaration of Helsinki. Data were analyzed by 1-way or 2-way ANOVA using Prism software (GraphPad), and *P* < .03 considered significant. All data are presented as mean ± SEM.

## 3 Results

### 3.1 Inhibition of TNIK impairs platelet function

**Figure 1:**
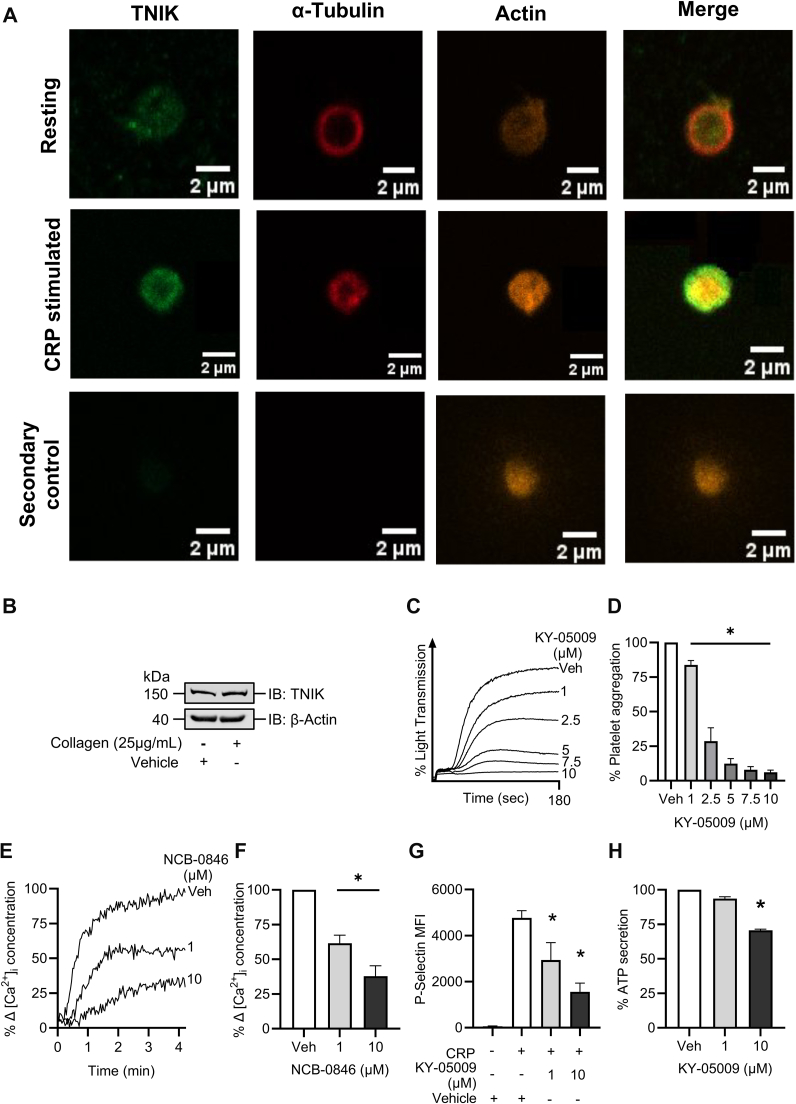
TRAF2 and NCK interacting kinase (TNIK) is present in human platelets and is important for platelet function. (A) The localization of TNIK in resting or activated (collagen-related peptide [CRP], 1 μg/mL) platelets. Platelets treated with integrilin (4 μM), were permeabilized (TritonX-100, 0.2% v/v) and visualized using confocal microscopy. TNIK (green), α-tubulin (red), and F-actin (orange) were stained using anti-TNIK (1:100 dilution) and anti–α-tubulin (1:200 dilution) antibodies, respectively, and visualized using anti-rabbit highly cross absorbed IgG Alexa Fluor 488 (1:1000 dilution), anti-mouse IgG Alexa Fluor 647 (1:250 dilution) secondary antibodies and Alexa Fluor 568 Phalloidin actin stain. For negative controls, primary antibodies were omitted, but phalloidin added for cell visualization and secondary antibody staining evaluated. Images were captured using a Zeiss LSM980 confocal microscope using a Plan-Apochromat 63X/1.4 oil differential interference contrast (DIC) objective. (B) Immunoblot showing presence of TNIK in platelet lysates. Representative (C) and mean (D) aggregation traces of platelets (4 × 10^8^ cells/mL) treated with KY-05009 (1-10 μM) or vehicle (0.5% v/v dimethylsulfoxide [DMSO]) for 5 minutes prior to stimulation with collagen (2.5 μg/mL). (E) Calcium mobilization in FURA-8 AM loaded (0.5 μM) platelets (4 × 10^8^ cells/mL) treated with vehicle (DMSO, 0.5% v/v) or NCB-0846 (1-10 μM) stimulated with CRP (0.5 μg/mL). (F) Mean calcium mobilization data. (G) P-selectin median fluorescence intensity values from platelets (2 × 10^8^ cells/mL) stimulated with CRP (0.5 μg/mL) in the presence of vehicle (DMSO, 0.5% v/v) or KY-05009 (1-10 μM). Platelets were gated and 10,000 events were recorded. (H) Mean adenosine triphosphate release from platelets (relative to vehicle; 4 × 10^8^ cells/mL) preincubated with vehicle (DMSO, 0.5% v/v) or KY-05009 (1-10 μM) before stimulation with CRP (1.0 μg/mL); *n* = 3. Data presented as mean ± SEM. All data analyzed by 1-way ANOVA; ∗*P* < .03.

**Supplementary Figure 1:**
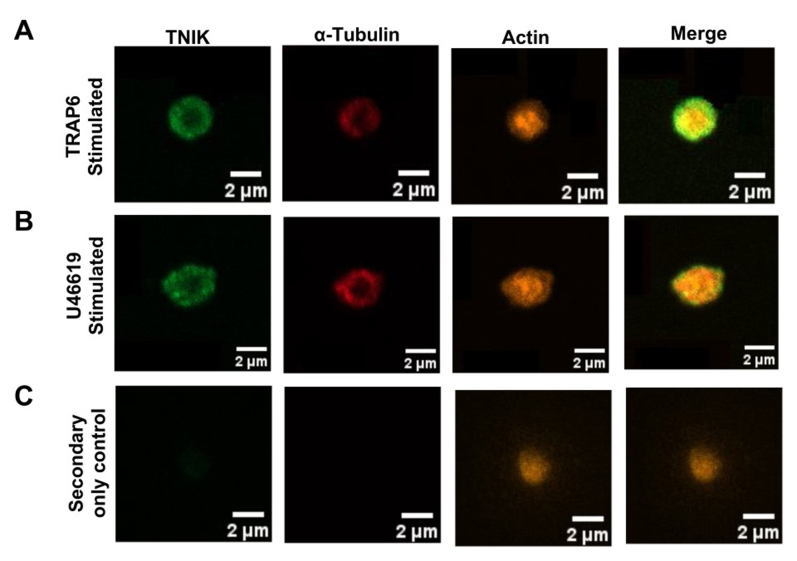
**TNIK is present in platelets and translocates to the cytoskeleton following platelet activation.** Shows the localisation of TNIK in resting or (A) TRAP-6 (10 μM) stimulated or (B) U46619 (5 μM) stimulated platelets. Platelets treated with integrilin (4 μM), were permeabilised (Triton X-100, 0.2% v/v) and visualised using confocal microscopy. TNIK (green), α-Tubulin (red) and F-actin (orange) were stained using anti-TNIK (1:100 dilution) and anti-α-Tubulin (1:200 dilution) antibodies respectively and visualised using anti-rabbit highly cross absorbed IgG Alexa Fluor 488 (1:1000 dilution), anti-mouse IgG Alexa Fluor 647 (1:250 dilution) secondary antibodies and Alexa Fluor 568 Phalloidin actin stain. (C) for negative controls, primary antibodies were omitted, but phalloidin added for cell visualisation and secondary antibodies staining evaluated. Images were captured using a Zeiss LSM980 confocal microscope using a Plan-Apochromat 63X/1.4 oil DIC objective. n=3.

In many cell types, TNIK has been recognized as an important cytoskeletal regulator implicated in cell motility [2] and neuronal dendrite development [5,28]. Proteomic analyses have identified TNIK to be present in platelets, but no further exploration at a protein level, has been conducted [[22], [23], [24], [25]]. In this study, the presence and localization of TNIK in platelets in both resting and stimulated conditions was explored using confocal microscopy. In resting platelets, TNIK is localized throughout the platelet cytoplasm and showed a distinct distribution when compared with the distribution of the α-tubulin cytoskeletal band and actin. Upon stimulation with CRP, TRAP-6, and U46619, a pool of TNIK remains in the cytoplasm, but as a result of activation, some TNIK appears to translocate to the cytoskeletal ring (Figure 1A). Pearson's correlation coefficient confirmed these activation-dependent changes in localization. Colocalization to α-tubulin increased following platelet CRP stimulation (resting, 0.2532 ± 0.02), CRP stimulated (0.7066 ± 0.03; *P* = .0003). A similar activation-dependent change in localization toward actin was observed (resting platelets, 0.2978 ± 0.02), which increased in CRP-stimulated platelets (0.7166 ± 0.03; *P* = .005). Similarly, we observed an increase in colocalization between TNIK and Tubulin following U46619 simulation (0.6413 ± 0.04; *P* = .0021) or TRAP-6 stimulation (0.606 ± 0.03; *P* = .0006) and between TNIK and actin following stimulation (U46619, 0.6804 ± 0.08; *P* = .006) and TRAP-6 (0.6382 ± 0.04; *P* = .0009) (Supplementary Figure 1A–C). TNIK protein expression in platelets was also confirmed by immunoblot of resting and activated platelets and levels did not change following platelet activation (Figure 1B), supporting the idea that TNIK redistributes upon platelet activation rather than increasing copy number.

**Supplementary Figure 2:**
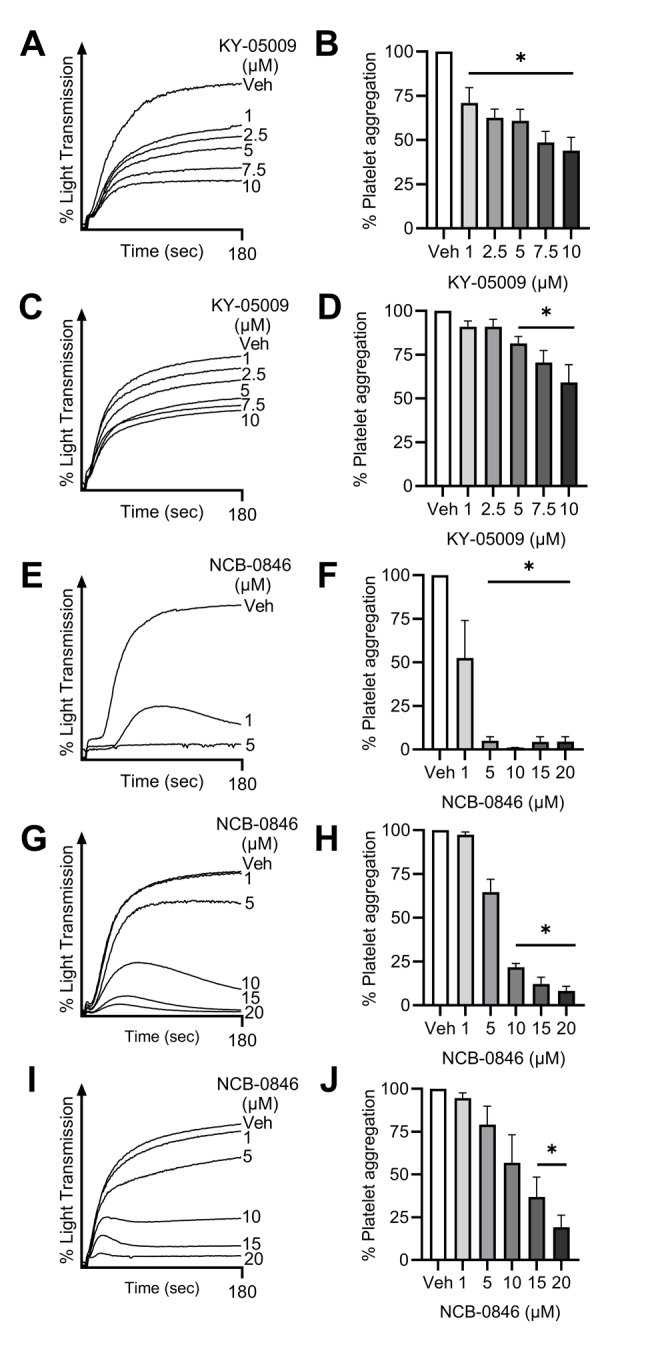
**TNIK inhibition significantly reduces aggregatory responses.** Representative (A) and mean (B) aggregation traces of platelets (4x10^8^ cells/mL) treated with KY-05009 (1-10 μM) or vehicle (0.5% v/v DMSO) for 5 minutes prior to stimulation with TRAP-6 (5μM). Representative (C) and mean (D) aggregation traces of platelets (4x10^8^ cells/mL) treated with KY-05009 (1-10 μM) or vehicle (0.5% v/v DMSO) for 5 minutes prior to stimulation with U46619 (1 μM). Representative (E) and mean (F) aggregation traces of platelets (4X10^8^ cells/mL) treated with NCB-0846 (1-20 μM) or vehicle (0.5% v/v DMSO) for 5 minutes prior to stimulation with collagen (2.5 μg/mL). Representative (G) and mean (H) aggregation traces of platelets (4x10^8^ cells/mL) treated with NCB-0846 (1-20 μM) or vehicle (0.5% v/v DMSO) for 5 minutes prior to stimulation with TRAP-6 (5 μM). Representative (I) and mean (J) aggregation traces of platelets (4x10^8^ cells/mL) treated with NCB-0846 (1-20 μM) or vehicle (0.5% v/v DMSO) for 5 minutes prior to stimulation with U46619 (1 μM). n=3. Data presented as mean ± SEM. Data analysed by one-way ANOVA. ∗ p<0.03

To investigate a potential role for TNIK in platelet function, human platelets were preincubated with TNIK inhibitor KY-05009 and platelet aggregation was measured using aggregometry. Platelets treated with KY-05009 (1-10 μM) exhibited a significant, dose-dependent reduction in aggregation in response to collagen (Figure 1C, D). At the highest concentration tested, KY-05009 (10 μM) decreased aggregation by 93% (±1.6 %). KY-05009 had a calculated IC_50_ of 1.8 μM in collagen-stimulated platelets. Aggregation in response to TRAP-6 and the TxA2 mimetic U46619 were also significantly reduced following KY-05009 treatment (Supplementary Figure 2A–D). The effects of TNIK inhibition on platelet aggregation were also validated with a structurally distinct TNIK inhibitor NCB-0846, which yielded a similar, dose-dependent reduction in aggregation for all agonists tested (Supplementary Figure 2E–J).

Platelets were loaded with FURA-8 AM to investigate a potential role of TNIK signaling in the control of calcium mobilization from intracellular stores. As KY-05009 fluoresces in the emission range of FURA-8 AM, TNIK inhibition was achieved using NCB-0846. Calcium mobilization was reduced significantly (62.5% ± 7.5) following NCB-0846 (10 μM) treatment (Figure 1E, F).

The effects of TNIK inhibition on α-granule secretion was investigated using flow cytometry to measure P-selectin expression on the surface of platelets. Resting, vehicle-treated platelets expressed low levels of P-selectin on their surface (median fluorescence intensity [MFI], 63.8 ± 15.7), which dramatically increased (MFI, 4772 ± 306.7), following activation with CRP. Activated platelets treated with KY-05009 (10 μM) exhibited a 70.0% (±5.9%) reduction in P-selectin surface expression (MFI, 1559 ± 378.8) compared with vehicle-treated, activated platelets (Figure 1G).

The effects of TNIK inhibition on dense granule secretion was measured by ATP release. In platelets treated with KY-05009 (10 μM), a significant reduction (38.6% ± 8.7%) in ATP release compared with the vehicle was observed (Figure 1H). These results suggest TNIK is a novel regulator of platelet aggregation, calcium mobilization, and granule secretion, all of which are facilitated by dynamic reorganization of the platelet cytoskeleton [[29], [30], [31]].

### 3.2 Inhibition of TNIK impairs platelet tethering and spreading on immobilized fibrinogen

Since TNIK has been shown to regulate a host of actin-binding proteins in other cell types [2,3,5] we sought to investigate if TNIK modulates the platelet actin cytoskeleton remodeling that underpins shape change and spreading. Resting (unstimulated) platelets were adhered and spread on immobilized fibrinogen in the presence of vehicle (DMSO, 0.5% v/v) or KY-05009 (10 μM).

**Figure 2:**
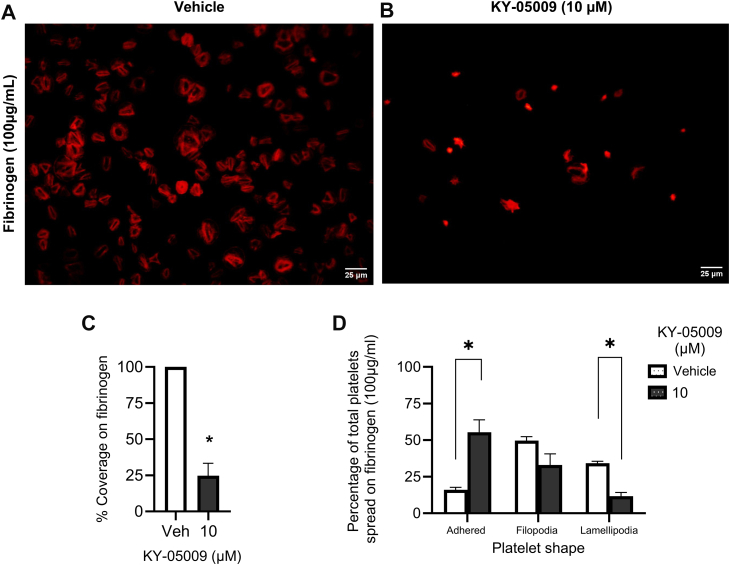
KY-05009 impairs platelet tethering to immobilized fibrinogen. Adhesion of (A) vehicle (0.5% v/v dimethylsulfoxide [DMSO]) or (B) KY-05009 (10 μM)-treated platelets (4 × 10^7^ cells/mL) to fibrinogen (100 μg/mL). F-actin was stained with Alexa Fluor 568 phalloidin (1:100 dilution). Platelets were visualized with a Nikon Eclipse Ts2 Fl microscope using a 40X/0.65 objective. Representative images are shown: (C) percentage platelet coverage and (D) mean data; *n* = 4. Data are presented as mean ± SEM. Data analyzed by 1-way ANOVA; ∗*P* < .03.

**Supplementary Figure 3:**
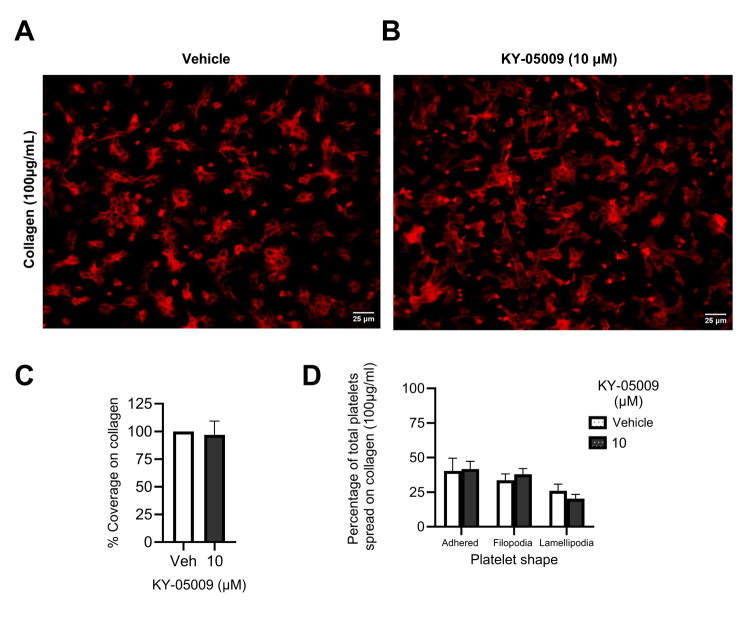
**TNIK inhibition does not impair platelet adhesion and spreading on immobilised collagen.** Adhesion of (A) vehicle (0.5% v/v DMSO) or (B) KY-05009 (10 μM) treated platelets to collagen (100 μg/mL). F-actin was stained with Alexa Fluor 568 Phalloidin (1:100 dilution). Platelets were visualised with a Nikon Eclipse Ts2 Fl microscope using a 40X/0.65 objective. Representative images are shown, (C) is percentage platelet coverage and (D) is mean data. n=4. Data presented as mean ± SEM. Data analysed by one-way ANOVA.

Compared with vehicle, KY-05009 treatment diminished platelet spreading on fibrinogen, the primary ligand for α_IIb_β_3_ (Figure 2A–C). KY-05009–treated platelets exhibited a trend toward lower filopodia formation than the vehicle-treated platelets, while lamellipodia formation in KY-05009–treated platelets was reduced by 64.6% (±2.5%). To quantify platelet adhesion, we measured percentage coverage of platelets on fibrinogen; this was also impaired by 75.3% (±8.6%) relative to vehicle, suggesting that inhibition of TNIK impairs both initial platelet adhesion and then subsequent spreading onto immobilized fibrinogen. TNIK inhibition did not significantly affect platelet adhesion and spreading on immobilized collagen (Supplementary Figure 3A–C), suggesting TNIK is not involved in integrin α_2_β_1_-mediated signaling processes.

### 3.3 TNIK inhibition impairs platelet clot retraction by reducing integrin α_IIb_β_3_ activation

**Figure 3:**
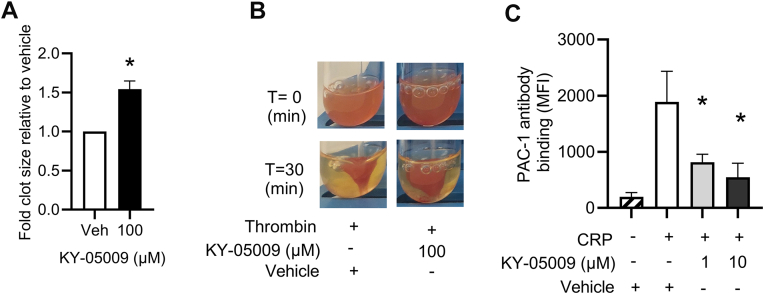
TRAF2 and NCK interacting kinase (TNIK) inhibiton impairs clot retraction and integrin α_IIb_β_3_ activation. Platelet-rich plasma (PRP) was incubated for 5 minutes in the presence of vehicle (dimethylsulfoxide [DMSO], 1% v/v) or KY-05009 (100 μM) prior to stimulation of clot formation with thrombin (1 unit/mL). (A) Clot size after 30 minutes relative to vehicle. (B) Representative images of clot retraction after 30 minutes. Integrin α_IIb_β_3_ activation was investigated through monitoring PAC-1 antibody binding using flow cytometry (C). Platelets (2 × 10^8^ cells/mL) were stimulated with CRP (0.5 μg/mL) in the presence of vehicle (DMSO, 0.5% v/v) or KY-05009 (1-10 μM) and 10,000 gated events were recorded. Raw median fluorescence intensity (MFI) values are shown (C); *n* = 3. Data are presented as mean ± SEM. Data analyzed by 1-way ANOVA; ∗*P* < .03.

**Supplementary Figure 4:**
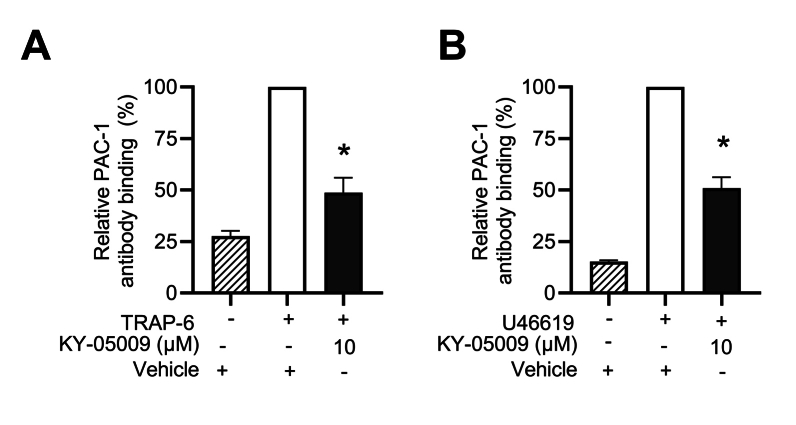
TNIK inhibition significantly impairs PAC-1 antibody binding in TRAP-6 and U46619 stimulated platelets. PAC-1 antibody binding to activated integrin αIIbβ3 was monitored in washed platelets (2 × 10^8^ cells/mL) treated with vehicle (DMSO, 0.5% v/v) or KY-05009 (1, 10 μM) and stimulated with (A) TRAP-6 (5 μM) or (B) U46619 (1 μM). Platelets were gated and 10,000 events were recorded. Data is mean ± SEM and analysed by one-way ANOVA. ∗ *P* < .03, *n* ≥ 3.

TNIK inhibition led to impaired platelet adhesion and spreading on fibrinogen (Figure 2A–C), so, to investigate whether this was due to TNIK regulating platelet shape change through integrin α_IIb_β_3_ activation and associated outside-in signaling, we monitored clot retraction. Following KY-05009 (100 μM) treatment clot retraction was defective with relative clot size increasing by 54.3% (±0.1%) (Figure 3A, B). During platelet activation, integrin α_IIb_β_3_ switches from a low-affinity conformation with limited fibrinogen binding to a high-affinity binding state, which is essential for thrombus formation [32,33]. Since TNIK inhibition impaired clot retraction, we explored whether this could be due to an impairment in integrin α_IIb_β_3_ activation. Resting, vehicle-treated platelets showed low levels of integrin α_IIb_β_3_ activation as measured by PAC-1 antibody binding (MFI, 199.2 ± 76), which increased substantially (MFI, 1889 ± 545.6) following stimulation with CRP. In KY-05009 (10 μM)-treated platelets, PAC-1 antibody binding was significantly reduced by 71.5% (±5.5%; MFI, 545.3 ± 251.6) (Figure 3C), compared with activated control samples. Platelets treated with KY-05009 (10 μM) prior to TRAP-6 stimulation, exhibited a 51.3% (±7.3%; MFI, 387.8 ± 90.3) reduction in PAC-1 antibody binding compared with the vehicle-treated, TRAP-6-stimulated control (MFI, 967.8 ± 244.4). Similarly, KY-05009–treated platelets stimulated with U46619 exhibited a significant reduction in PAC-1 antibody binding 49.1% (±5.3%; MFI, 908.3 ± 316.5) compared with vehicle-treated U46619-stimulated platelets (MFI, 2016.0 ± 419.1) (Supplementary Figure 4A, B). Taken together, these findings indicate that TNIK regulates integrin α_IIb_β_3_ conformational changes, leading to reduced inside-out signaling–mediated activation of integrin αIIbβ3, but may also play a role in the regulation of outside-in signaling–mediated integrin α_IIb_β_3_ activation essential for platelet adhesion to and spreading on immobilized fibrinogen.

### 3.4 TNIK modulates integrin α_IIb_β_3_ activation via the PKC signaling pathway

**Figure 4:**
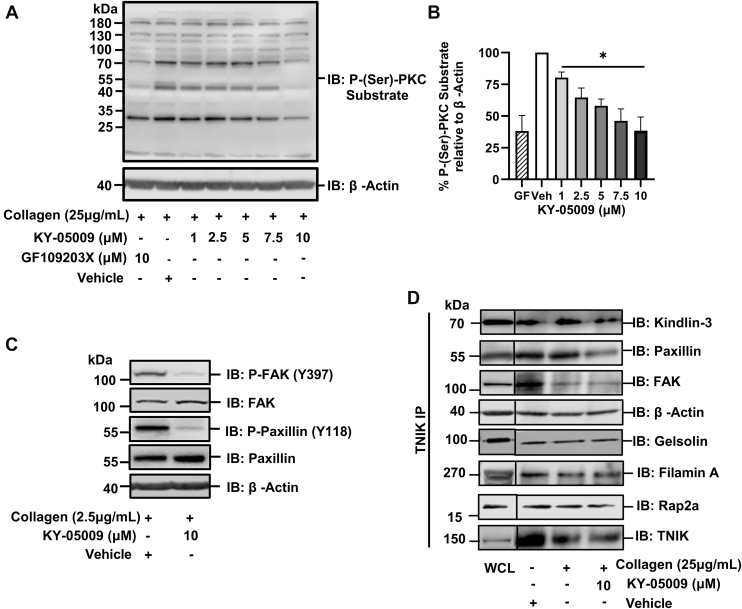
TRAF2 and NCK interacting kinase (TNIK) inhibition leads to reduced phosphorylation of PKC substrates and the integrin α_IIb_β_3_ mediators focal adhesion kinase (FAK) and paxillin. Non-aggregating platelets were prepared in the presence of indomethacin (10 μM), EGTA (1 mM), and apyrase (2 units/mL) to a cell density of 4 × 10^8^ cells/mL. (A) Serine phosphorylation of PKC substrates in platelets stimulated with collagen (25 μg/mL) for 3 minutes in the presence of vehicle (dimethylsulfoxide [DMSO]; 0.5% v/v) or KY-05009 (1-10μM) ± PKC inhibitor GF 109203X (10 μM). (B) mean p-(ser) PKC substrate phosphorylation data normalized to β-actin. (C) Immunoblot analysis of FAK and paxillin phosphorylation in platelets stimulated with collagen (25 μg/mL). For immunoprecipitation, platelets were prepared in non-aggregating conditions and resuspended to a density of 8 × 10^8^ cells/mL. Resting and collagen-stimulated (25 μg/mL) platelets were lysed in 1% (v/v) NP40 lysis buffer before TNIK was immunoprecipitated using 1 μg of TNIK antibody. Immuno-protein complexes were captured with protein A/G beads. Beads were washed with 0.5% (v/v) NP40 buffer before being separated by sodium dodecyl sulfate–polyacrylamide gel electrophoresis. (D) TNIK immunoprecipitates were probed using Kindlin-3, paxillin, FAK, actin, gelsolin, Rap2a, filamin A and TNIK-specific primary antibodies (all 1:1000 dilution) and visualized with horseradish peroxidase (HRP)-conjugated secondary antibodies; *n* = 3. Data presented as mean ± SEM. Data analyzed by 1-way ANOVA; ∗*P* < .03.

TNIK inhibition diminished platelet aggregation, granular secretion, calcium mobilization, and integrin α_IIb_β_3_ activation following stimulation; in platelets, PKC signaling orchestrates many of these processes. To determine whether the observed impairment of platelet function was due to convergence of TNIK signaling with PKC pathways, PKC substrate phosphorylation was measured in collagen-stimulated platelets (3-minute stimulation). Following TNIK inhibition alongside a PKC inhibitor GF109203X (10 μM) [34], KY-05009 treatment reduced PKC substrate phosphorylation in a dose-dependent manner. At the highest dose tested (10 μM), we saw a 52.3% (±10.1%) reduction in phosphorylation of PKC substrates (Figure 4A, B). To date, TNIK has not been shown to directly interact with PKC, but in other cell types, TNIK has been shown to mediate cytoskeletal changes through control of FAK [12]. FAK is activated by autophosphorylation at Y397. We observe a reduction in FAK phosphorylation at Y397 following TNIK inhibition (Figure 4C). Since FAK activity is dependent on upstream PKC signaling [35], it is feasible that TNIK acts as a modulator of PKC signaling or is an intermediary between PKC and FAK.

FAK is also regulated by the actin-binding protein paxillin through an undetermined mechanism. Following activation by phosphorylation at Y118, paxillin works alongside focal adhesion proteins to facilitate cell motility and shape change [36]. We observe a reduction in paxillin phosphorylation in KY-05009–treated platelets. Furthermore, paxillin interacts with kindlin-3 to support the activation of integrin α_IIb_β_3_. Kindlin-3 is phosphorylated at Y482 and S484, by PKC, which is essential for integrin α_IIb_β_3_-mediated signaling [37]. Since there are no commercially available P–kindlin-3 (Y482/S484) antibodies available, it is not currently possible to determine whether TNIK modulates kindlin-3 phosphorylation. To explore whether TNIK localizes with these proteins at sites of focal adhesion, we immunoprecipitated TNIK from platelet lysates and observed an interaction between TNIK, actin, FAK, paxillin, kindlin-3, Rap2a, gelsolin and filamin A (Figure 4D). These results position TNIK as a localized component of the focal adhesions that form in proximity to integrin αIIbβ3, which regulates platelet shape change by modulating activation of essential components of focal adhesion complexes, and by interaction with the actin regulatory proteins gelsolin, Rap2a and filamin A.

### 3.5 TNIK inhibition significantly reduces thrombus formation under arterial shear conditions

**Figure 5:**
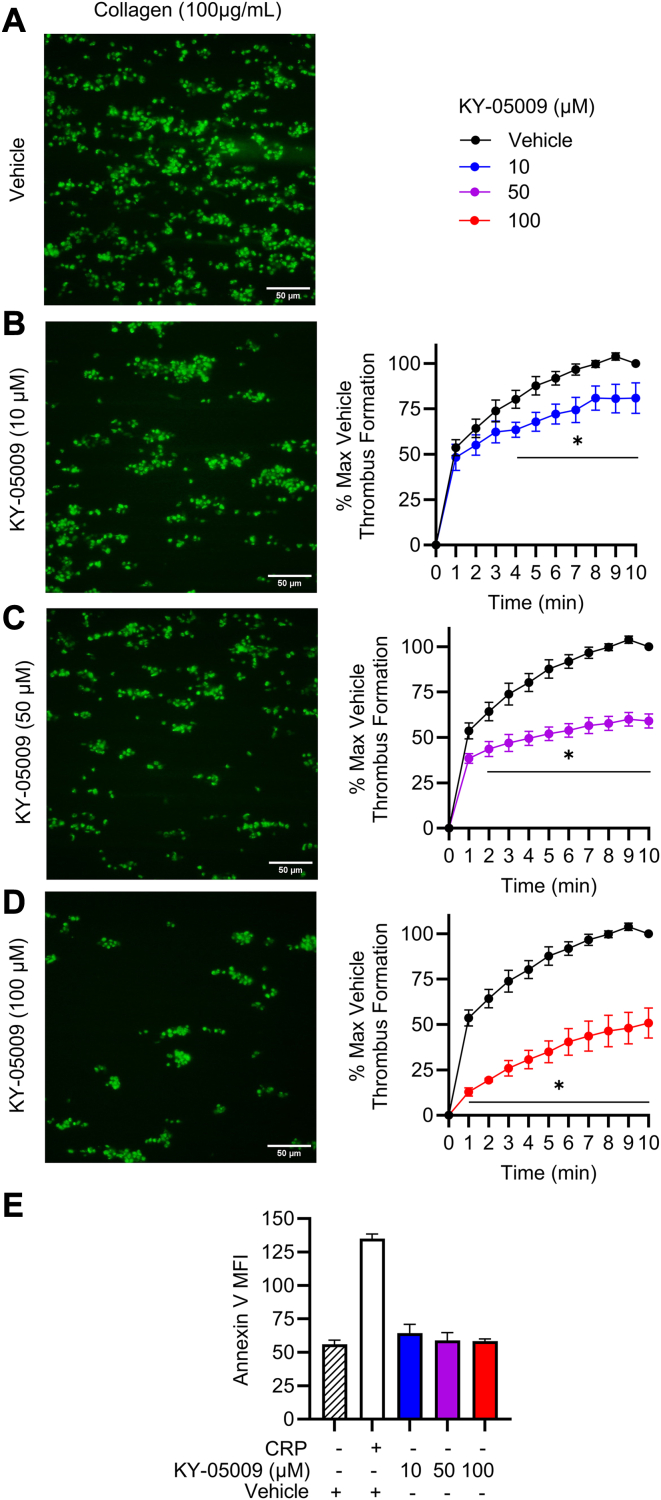
KY-05009 treatment attenuates thrombus formation under arterial shear stress. DIOC6 labeled whole blood was treated with (A) vehicle (DMSO, 1% v/v), (B) KY-05009 (10 μM), (C) KY-05009 (50 μM), or (D) KY-05009 (100 μM) and perfused over collagen coated (100μg/mL) Vena8 Fluoro+ Biochips using a Cellix perfusion system at arterial sheer stress (20 dynes/cm^2^) for 10 minutes. Thrombus formation was determined by monitoring the change in fluorescence intensity observed over recorded intervals. Representative images were taken using a Nikon Eclipse Ts2 Fl microscope with a 40X/0.65 objective. The fluorescence intensity of the formed thrombi were normalized to the maximum fluorescence intensity value of the vehicle after 10 minutes and expressed as a percentage; *n* = 6. Decimated averages at each time point are presented ± SEM. (E) Annexin V binding to platelets treated with vehicle (DMSO, 1% v/v) or KY-005009 (10-100 μM). Platelets were gated and 10 000 events were recorded. Mean ± SEM of raw median fluorescence intensity data are presented; *n* = 3. Thrombus formation data analyzed by 2-way ANOVA; flow cytometry data analyzed by 1-way ANOVA; ∗*P* < .03.

Perfusion assays were used to determine whether TNIK plays a role in the regulation of thrombus formation *in vitro*, under physiological shear. DIOC6-labeled whole blood was perfused over microfluidic channels in the presence of either vehicle (DMSO, 1% v/v) or KY-05009 (10, 50, or 100 μM). TNIK inhibition substantially impaired integrin α_IIb_β_3_-mediated thrombus formation across all tested doses (Figure 5A–C). We observed an impairment in the early stages (timepoints under 4 minutes) of thrombus formation following TNIK inhibition with 10 μM of KY-05009, while higher doses (50 and 100 μM KY-05009) further impaired thrombus formation at earlier timepoints. Supportive of the functional defects observed in Figure 1, across all tested doses, KY-05009 significantly impaired thrombus formation under arterial shear conditions. After 10 minutes, at the lowest dose tested (10 μM KY-05009), we observed a reduction in thrombus formation of 19.2% (±8.4%), at 50 μM KY-05009, thrombus formation was 41.0% (±3.9%) lower than the control and a 55.9% (±5.8%) reduction following treatment with 100 μM KY-05009 was observed at the endpoint. To ensure these observations were not due to drug-induced platelet cytotoxicity, we monitored fluorescent annexin V surface binding by flow cytometry as a marker of platelet apoptosis [38]. Resting, vehicle-treated (DMSO, 1% v/v) platelets showed low annexin V binding (MFI, 56.0 ± 3.1), which increased (MFI, 135.0 ± 3.5) when platelets were robustly stimulated with CRP. We observed no significant difference or increase in annexin V binding in platelets incubated with varying doses of KY-05009 (highest concentration tested was 100 μM; MFI, 58.3 ± 1.8), which was comparable with the resting, vehicle-treated control levels (MFI, 56.0 ± 3.1). These results indicate KY-05009 does not induce platelet cytotoxicity or apoptosis.

## 4 Discussion

Platelet kinases play an integral role in potentiating and sustaining proaggregatory signaling. Recent advances in proteomics have expanded our knowledge of the platelet kinome, but many of the kinases identified have yet to be extensively investigated to elucidate their role in thrombosis and hemostasis. In this study, we characterized a novel kinase, TNIK, which we hypothesized is involved in the regulation of platelet signaling through interactions with key cytoskeletal regulators to facilitate platelet shape change. Previously, proteomic studies exploring novel members of the platelet kinome identified TNIK in platelets [[22], [23], [24], [25]] with human platelets expressing around 2200 copies of TNIK per platelet [25]. However, to date, TNIK’s function in platelets had not been fully investigated. We first confirmed the presence and localization of TNIK in platelets with immunoblotting and confocal microscopy, which revealed a partial translocation of TNIK to the cytoskeleton following platelet activation.

TNIK activity was inhibited using the structurally distinct inhibitors, KY-05009 and NCB-0846, to investigate a potential role of TNIK in the regulation of platelet function. The kinase domain of TNIK contains N and C lobes connected by a hinge region, which functions as an ATP-binding site. Commonly, TNIK inhibitors impair TNIK kinase function by restricting the movement of the N and C lobes. Under normal conditions, following ATP binding, the N lobe of TNIK switches from an inactive (open) configuration to an active (closed) configuration [39]. KY-05009 and NCB-0846 both form 2 hydrogen bonds with Cys108 in the hinge region, to impair the kinase activity of TNIK [40].

Inhibition of TNIK in human platelets resulted in a significant reduction in hallmarks of platelet inside-out signaling. Reduction in platelet aggregation and α-granule secretion could, in part, be explained by the reduction in the release of secondary mediator substances from dense granules. Alternatively, reduced platelet function could be due to the reduction in calcium mobilization also seen following TNIK inhibition. Indeed, modulation of calcium levels facilitates phosphorylation and activation of the contractile protein myosin light chain, which is important for platelet shape change and thrombus formation [41,42] through its interactions with kindlin-3 and integrin α_IIb_β_3_ [43]. The data presented in this study show kindlin-3 to be a binding partner of TNIK and integrin α_IIb_β_3_ activation to be regulated by TNIK-mediated signaling suggesting that TNIK can participate in both early (granule release) and later (integrin-related) platelet signaling events.

**Figure 6:**
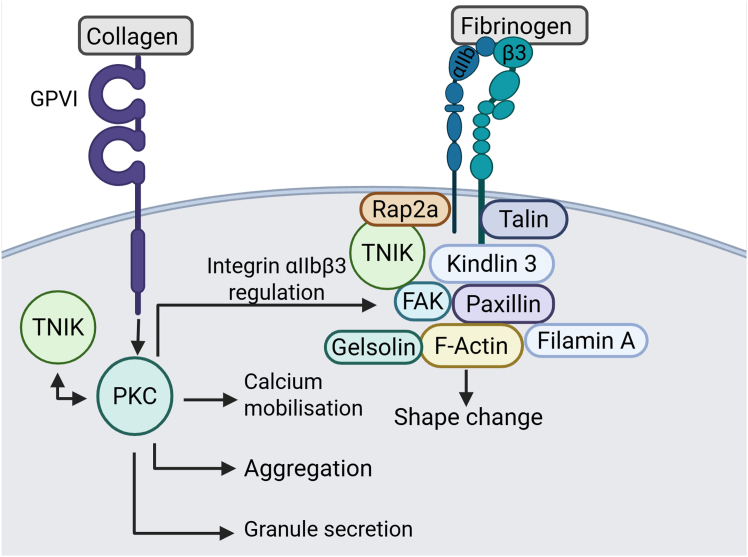
Schematic showing the roles of TRAF2 and NCK interacting kinase (TNIK) in platelets. In human platelets, TNIK inhibition impaired platelet aggregation, calcium mobilization, granule secretion, and PKC signaling. TNIK is a positive regulator of integrin α_IIb_β_3_–mediated cytoskeletal changes due to direct association with effectors of the focal adhesion kinase complex and the actin cytoskeleton. Created in BioRender. Bull, L. (2025) https://BioRender.com/v3ypbhf.

TNIK facilitates phosphorylation of actin-binding proteins to regulate morphological changes [2] and is known to interact with several actin-linker proteins, to regulate F-actin disassembly, thereby controlling neuronal structure, cell morphology, and migration [[3], [4], [5],^40^]. We observed a significant reduction in clot retraction, platelet adhesion, spreading, and thrombus formation following TNIK inhibition, suggesting that TNIK supports platelet inside-out signaling to regulate the activation of integrin α_IIb_β_3_. In these processes, activation of integrin α_IIb_β_3_ initiates the formation of focal adhesion sites and recruitment of effector proteins, which precede cytoskeletal changes. The relationship between the cytoskeletal regulator Rap2a and TNIK has been explored in a variety of cell types [3,5,18]. In this study, we confirmed, using immunoprecipitation that platelet TNIK interacts with Rap2a, while little is known about Rap2a in platelets, the related protein Rap1b is well documented as a facilitator of integrin activation [44], therefore, further investigation of this novel interaction could provide mechanistic insight into how TNIK inhibition results in impaired platelet shape change and reduced levels of integrin α_IIb_β_3_ activation. Similarly, FAK activity at focal adhesion sites, in other cells, has previously been shown to be regulated by TNIK [12]; in this study, we confirmed, in platelets, following TNIK inhibition, FAK phosphorylation is reduced. Since FAK activity is dependent on upstream PKC signals [24], it is possible that TNIK acts as a regulator of PKC signaling or is an intermediary between PKC signaling and focal adhesion site signals to the cytoskeleton [38]. FAK also associates with the adaptor protein paxillin, which positively regulates FAK autophosphorylation [45]. In focal adhesion complexes, paxillin interacts directly with kindlin-3 to support integrin α_IIb_β_3_ signaling [46,47]. Platelet TNIK inhibition resulted in a reduction in paxillin phosphorylation, which is consistent with observations in other cell types [40]. To confirm TNIK localizes to and regulates focal adhesion signaling, immunoprecipitation was used. TNIK coprecipitated with actin, FAK, paxillin, kindlin-3, Rap2a filamin A, and gelsolin, positioning it as a bridge between PKC, integrin α_IIb_β_3_, focal adhesion sites, and the actin cytoskeleton as illustrated in Figure 6.

In summary, we propose that TNIK is a novel regulator of integrin α_IIb_β_3_ activation and cytoskeletal changes in platelets that acts through positive regulation of platelet inside-out signaling. Since TNIK regulates thrombus formation under arterial shear conditions, future work should prioritize investigating the importance of TNIK in hemostasis and thrombosis *in vivo* and further interrogate the platelet TNIK interactome to address its potential as an antithrombotic target.
