# Supplementary material for: TRAF2 and NCK interacting kinase: a novel regulator of integrin αIIbβ3 signaling in platelets
Source: Res Pract Thromb Haemost. 2025 Oct 6;9(7):103204. doi: 10.1016/j.rpth.2025.103204 (PMC12616069; doi:10.1016/j.rpth.2025.103204)
Supplement: Supplementary Material [file mmc1.docx]

**Supplementary Figure 1: TNIK is present in platelets and translocates to the cytoskeleton following platelet activation.**

Shows the localisation of TNIK in resting or (A) TRAP-6 (10 μM) stimulated or (B) U46619 (5 μM) stimulated platelets. Platelets treated with integrilin (4 μM), were permeabilised (Triton-X-100, 0.2% v/v) and visualised using confocal microscopy. TNIK (green), α-Tubulin (red) and F-actin (orange) were stained using anti-TNIK (1:100 dilution) and anti-α-Tubulin (1:200 dilution) antibodies respectively and visualised using anti-rabbit highly cross absorbed IgG Alexa Fluor 488 (1:1000 dilution), anti-mouse IgG Alexa Fluor 647 (1:250 dilution) secondary antibodies and Alexa Fluor 568 Phalloidin actin stain. (C) for negative controls, primary antibodies were omitted, but phalloidin added for cell visualisation and secondary antibodies staining evaluated. Images were captured using a Zeiss LSM980 confocal microscope using a Plan-Apochromat 63X/1.4 oil DIC objective. n=3.

**Supplementary Figure 2: TNIK inhibition significantly reduces aggregatory responses**

Representative (A) and mean (B) aggregation traces of platelets (4x10^8^ cells/mL) treated with KY-05009 (1-10μM) or vehicle (0.5% v/v DMSO) for 5 minutes prior to stimulation with TRAP-6 (5 μM). Representative (C) and mean (D) aggregation traces of platelets (4x10^8^ cells/mL) treated with KY-05009 (1-10 μM) or vehicle (0.5% v/v DMSO) for 5 minutes prior to stimulation with U46619 (1 μM). Representative (E) and mean (F) aggregation traces of platelets (4X10^8^ cells/mL) treated with NCB-0846 (1-20 μM) or vehicle (0.5% v/v DMSO) for 5 minutes prior to stimulation with collagen (2.5 μg/mL). Representative (G) and mean (H) aggregation traces of platelets (4x10^8^ cells/mL) treated with NCB-0846 (1-20 μM) or vehicle (0.5% v/v DMSO) for 5 minutes prior to stimulation with TRAP-6 (5μM). Representative (I) and mean (J) aggregation traces of platelets (4x10^8^ cells/mL) treated with NCB-0846 (1-20 μM) or vehicle (0.5% v/v DMSO) for 5 minutes prior to stimulation with U46619 (1 μM). n=3. Data presented as mean ± SEM. Data analysed by one-way ANOVA. * p<0.03

**Supplementary Figure 3: TNIK inhibition does not impair platelet adhesion and spreading on immobilised collagen**

Adhesion of (A) vehicle (0.5% v/v DMSO) or (B) KY-05009 (10 μM) treated platelets to collagen (100 μg/mL). F-actin was stained with Alexa Fluor 568 Phalloidin (1:100 dilution). Platelets were visualised with a Nikon Eclipse Ts2 Fl microscope using a 40X/0.65 objective. Representative images are shown, (C) is percentage platelet coverage and (D) is mean data. n=4. Data presented as mean ± SEM. Data analysed by one-way ANOVA.

**Supplementary Figure 4: TNIK inhibition significantly impairs PAC-1 antibody binding in TRAP-6 and U46619 stimulated platelets**

PAC-1 antibody binding to activated integrin αIIbβ3 was monitored in washed platelets (2x10^8^ cells/mL) treated with vehicle (DMSO, 0.5% v/v) or KY-05009 (1, 10 μM) and stimulated with (A) TRAP-6 (5 μM) or (B) U46619 (1 μM). Platelets were gated and 10,000 events were recorded. Data is mean ± SEM and analysed by one-way ANOVA. * p<0.03, n≥3.
